# The Impact of Tobacco Cigarettes, Vaping Products and Tobacco Heating Products on Oxidative Stress

**DOI:** 10.3390/antiox11091829

**Published:** 2022-09-16

**Authors:** Rosalia Emma, Massimo Caruso, Davide Campagna, Roberta Pulvirenti, Giovanni Li Volti

**Affiliations:** 1 Department of Biomedical and Biotechnological Sciences, University of Catania, Via S. Sofia, 97, 95123 Catania, Italy; 2 Center of Excellence for the Acceleration of Harm Reduction (CoEHAR), University of Catania, Via S. Sofia, 89, 95123 Catania, Italy; 3 Department of Clinical and Experimental Medicine, University of Catania, Via S. Sofia, 97, 95123 Catania, Italy

**Keywords:** oxidative stress, tobacco, cigarette, airway diseases, harm reduction

## Abstract

Cells constantly produce oxidizing species because of their metabolic activity, which is counteracted by the continuous production of antioxidant species to maintain the homeostasis of the redox balance. A deviation from the metabolic steady state leads to a condition of oxidative stress. The source of oxidative species can be endogenous or exogenous. A major exogenous source of these species is tobacco smoking. Oxidative damage can be induced in cells by chemical species contained in smoke through the generation of pro-inflammatory compounds and the modulation of intracellular pro-inflammatory pathways, resulting in a pathological condition. Cessation of smoking reduces the morbidity and mortality associated with cigarette use. Next-generation products (NGPs), as alternatives to combustible cigarettes, such as electronic cigarettes (e-cig) and tobacco heating products (THPs), have been proposed as a harm reduction strategy to reduce the deleterious impacts of cigarette smoking. In this review, we examine the impact of tobacco smoke and MRPs on oxidative stress in different pathologies, including respiratory and cardiovascular diseases and tumors. The impact of tobacco cigarette smoke on oxidative stress signaling in human health is well established, whereas the safety profile of MRPs seems to be higher than tobacco cigarettes, but further, well-conceived, studies are needed to better understand the oxidative effects of these products with long-term exposure.

## 1. Introduction

**Figure 1:**
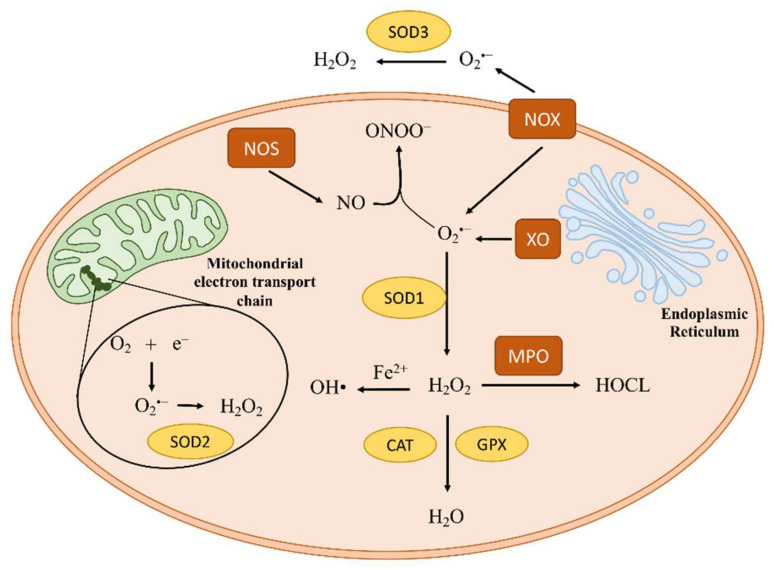
Endogenous sources of oxidants. Cellular ROS and RNS can be generated from different metabolic reactions, including mitochondrial electron transport and inflammation processes. Under physiological conditions, 0.2–2% of the electrons in the mitochondrial electron transport escape from the transport and interact with oxygen to produce superoxide (O_2_^•−^) or hydrogen peroxide (H_2_O_2_). Moreover, inflammatory processes contribute to the increase in ROS/RNS by the activation of numerous pro-oxidant enzymatic systems, such as NADPH oxidase (NOX), xanthine oxidase (XO), myeloperoxidase (MPO), and nitric oxide synthase (NOS). However, the presence of antioxidant enzymes, including superoxide dismutase (SOD), catalase (CAT), and glutathione peroxidase (GPX), contributes to maintaining the biological redox homeostasis.

The homeostasis of oxidation–reduction reactions is fundamental in biological processes. This biological redox homeostasis is maintained at a stable but non-equilibrium steady state, and different redox potentials are present at different cellular locations [1,2,3,4]. A deviation from the metabolic steady state leads to the concept of oxidative stress. “Oxidative stress” describes an imbalance between oxidants, such as reactive oxygen species (ROS) or reactive nitrogen species (RSN), and antioxidant species, in favor of oxidants [1]. There are two facets of reactive species: (i) physiological levels of oxidants are used for redox signaling and to control sophisticated biological mechanisms (oxidative eustress); (ii) on the other hand, a high concentration of oxidants causes damage to biomolecules and is harmful to cells (oxidative distress) [1,2,5]. ROS/RNS are reactive chemical species with one or more unpaired electrons in their outer orbital, making these molecules very unstable and able to initiate oxidation [5,6]. The sources of ROS/RNS can be endogenous or exogenous. Endogenous sources result from metabolic reactions, such as during mitochondrial electron transport or during inflammation processes, when numerous enzymatic systems are activated (e.g., NADPH oxidase, xanthine oxidase, myeloperoxidase, lipoxygenase, angiotensin II, and nitric oxide synthase) [5,6,7,8] (Figure 1). Meanwhile, the main exogenous sources of free radicals are environmental pollution and tobacco smoking [7].

**Figure 2:**
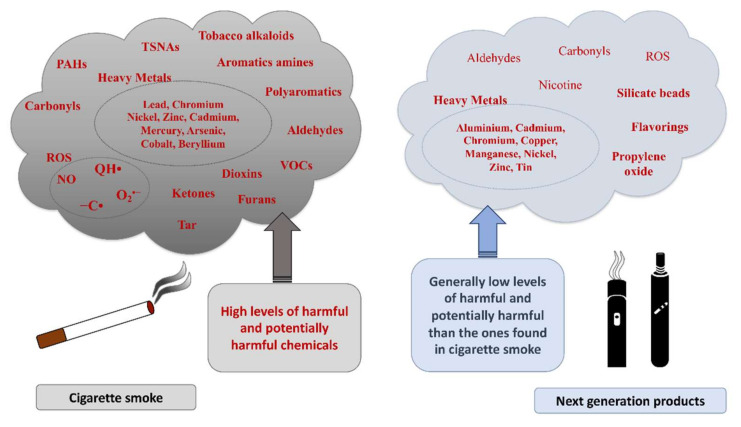
Chemical compounds generated by cigarette smoke and next-generation products (NGPs). Tobacco smoke is a complex mixture of thousands of different harmful and potentially harmful chemical species, including toxicants, carcinogens, and organic compounds (left panel). Aerosols generated by NGPs also contain harmful and potentially harmful compounds produced through the thermal decomposition of the solvents, but their quantity is generally lower compared to the ones found in cigarette smoke.

Tobacco smoke is a complex mixture of thousands of different chemical species (e.g., ROS/RNS, quinones, aldehydes, ketones, metals), which significantly contributes to enhanced oxidative stress (Figure 2): a single puff of cigarette smoke contains more than 10^15^ oxidants/free radicals [9,10,11], which exist in gas and tar phases. 

In addition to numerous ROS, epoxides, peroxynitrate, and NO are present in the gas phase, whereas the tar phase contains semiquinone, peroxides, hydroxyl radicals, hydrogen peroxide, and other organic compounds, which are involved in redox cycling with the generation of the superoxide anion [12,13]. Direct or indirect oxidative damage can be induced by these reactive chemical compounds, through the generation of pro-inflammatory compounds (chemokines, cytokines, prostaglandins, leukotrienes, isoprostanes) and the modulation of intracellular pro-inflammatory pathways (MAPK, NF-kB, AP-1, Keapl-Nrf2-ARE) [10,14,15,16]. Consequently, the inhalation of cigarette smoke contributes to the onset, maintenance, and progression of the inflammatory response, resulting in several so-called tobacco-related diseases (respiratory and cardiovascular diseases, tumors, etc.) [10,17,18,19]. Comparisons between tobacco smoke and electronic cigarette (e-cigarette) aerosol showed that the latter released lower levels of toxic compounds (e.g., formaldehyde, acetaldehyde, acrolein, and toluene) and ROS, but non-negligible levels of potential carcinogens, heavy metals, tin, silicate beads, flavoring, and propylene oxide derived from propylene glycol heating (e-cigarette solvent).

Cessation of smoking reduces the morbidity and mortality associated with cigarette use, and improves tobacco-related health consequences [20,21,22]. Therefore, rigorous efforts to control tobacco use, including increased taxation, enhanced educational programs and campaigns, prohibiting smoking in public areas, and the provision of smoking cessation services, have been implemented [21]. Nevertheless, cigarette smoking remains the most important preventable risk factor for morbidity and mortality worldwide, even though a reduction in smoking prevalence was observed in the past after the application of these measures [23]. Lower-risk alternatives to combustible cigarettes have been proposed as a harm reduction strategy to reduce the deleterious impacts of cigarette smoking [24]. This was the focus of the development of next-generation products (NGPs), including e-cigarettes and tobacco-heating products (THPs). E-cigarettes are electrical devices that heat and vaporize a liquid solution, containing propylene glycol (PG) and vegetal glycerol (VG) with or without nicotine, and, in most cases, flavorings. 

Meanwhile, THPs generate aerosols containing nicotine by heating a small element, similar to a small cigarette, including a tobacco plug into a hollow acetate tube, a polymer–film filter, a cellulose–acetate mouthpiece filter, and mouth-end papers, generally to referred as a heated tobacco product (HTP) [25], with reduced temperatures (up to 350 °C) that do not reach combustion (about 900 °C) [26].

The exponential growth and use of these products has generated an intensive debate about their public health impact, which has divided the scientific community into two groups: one group believes that NGPs could help smokers to quit cigarette consumption and to reduce its deleterious effects [27,28,29,30]; instead, the other group considers these products as an unsafe alternative to tobacco cigarettes, which could reverse the progress made by tobacco control measurements [31,32,33], bringing young people and future generations to nicotine addiction [34]. In this review, we examine the impact of tobacco smoke and NGPs on oxidative stress in different pathologies, including respiratory and cardiovascular diseases, tumors, and other possible health conditions related to smoking habits.

## 2. Oxidative Stress and Smoking/Vaping Related to Airway Diseases

**Figure 3:**
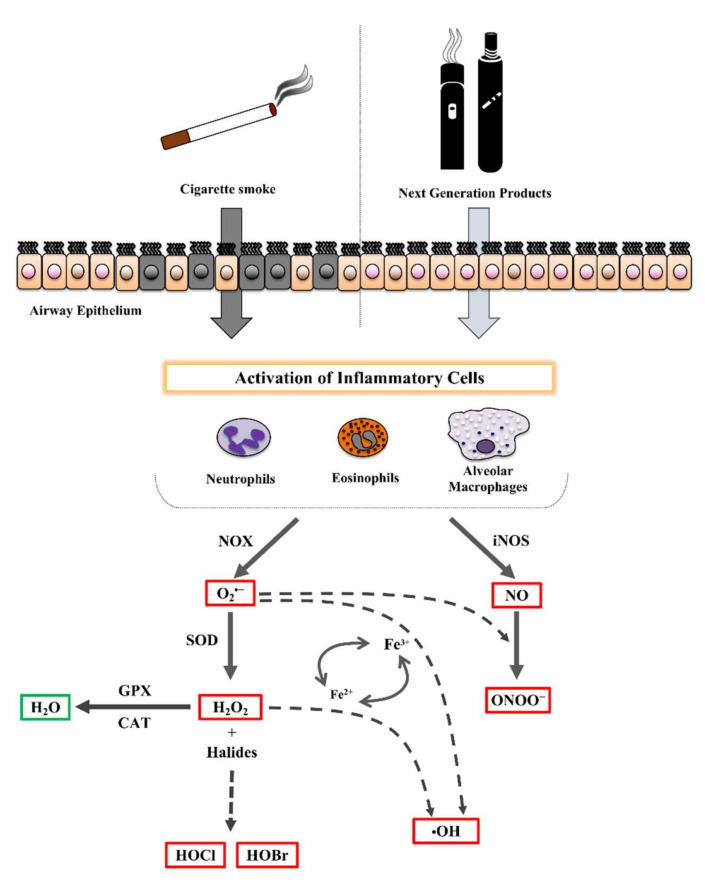
Generation of inflammatory response in the lung. Cigarette smoke induces cytotoxicity in the airway, promoting the persistence of activated pro-inflammatory cells (neutrophils, eosinophils, and macrophages) and their endogenous production of reactive oxygen species (ROS). The aerosols of next-generation products (NGPs) seem to display lower cytotoxicity than cigarette smoke. However, chemicals generated from NGPs could promote the activation of inflammatory cells, although the literature data are conflicting to date. Black cells = necrotic cells; cells with brown nuclei = damaged cells; cells with pink nuclei = normal cells; O_2_^•−^ = superoxide anion; H_2_O_2_ = hydrogen peroxide; HOBr = hypobromous acid; HOCl = hypochlorous acid; NO = nitric oxide; RSN = reactive nitrogen species; ^•^OH = hydroxyl radical; Fe^2+^ = ferrous ion; NOX = NADPH oxidase; SOD = superoxide dismutase; GPX = glutathione peroxidase; CAT = catalase; iNOS = inducible nitric oxide synthase.

Due to their anatomy and function, the airways are constantly exposed not only to atmospheric oxygen, but also to environmental oxidants (ozone, nitrogen dioxide, diesel exhaust, and cigarette smoke), thus making them susceptible to oxidative injury [9,35]. In addition, the recruitment of inflammatory cells (neutrophils, eosinophils, alveolar macrophages, etc.) after exposure to trigger factors (pathogens or irritants) contributes to enhanced oxidative stress, as observed in chronic obstructive pulmonary disease and asthma [36,37]. Unlike the environmentally derived ROS/RNS, cellular-derived ROS/RNS are produced by enzymatic systems, including NADPH oxidase (NOX), mitochondrial respiration, xanthine/xanthine oxidase, and nitric oxide synthase (NOS) [36,38]. An overview of these mechanisms is shown in Figure 3. The first oxidant molecule released by inflammatory cells is the superoxide anion (O_2_^•−^), mainly produced through the NOX pathway [39]. Moreover, mechanical and environmental stimuli can cause mitochondrial dysfunction in the airway epithelial cells, which contributes to the production of O_2_^•−^ [40]. Normally, the O_2_^•−^ is quickly converted to hydrogen peroxide (H_2_O_2_) by superoxide dismutase enzymes (SOD) [41], and H_2_O_2_ is transformed into H_2_O by two antioxidant enzymes: catalase (CAT) and glutathione peroxidase (GPX) [9]. However, H_2_O_2_ can react with Fe^2+^ in a non-enzymatic manner (Fenton reaction), resulting in the oxidation of Fe^2+^ to Fe^3+^ and generation of the hydroxyl radical (^•^OH). Moreover, the O_2_^•−^ radical can react with Fe^3+^ with the generation of ^•^OH and the regeneration of Fe^2+^ through the Haber–Weiss reaction [38,42]. The H_2_O_2_, not converted into H_2_O, can also interact with halides to form hypobromous acid (HBrO) or hypochlorous acid (HClO) through the action of heme peroxidase, eosinophil peroxidase (EPX), and myeloperoxidase (MPO) [43]. Nitric oxide (NO) is the other free radical produced by activated inflammatory cells through the degradation of L-arginine to L-citrulline by the inducible and calcium-independent isoform of NOS enzymes (iNOS) [44,45]. The up-regulation of iNOS leads to increased levels of NO, which can react with the O_2_^•−^ radical to form a highly reactive peroxynitrite molecule (ONOO^−^) [36].

Due to the constant exposure to external and internal oxidative compounds, the airways are the only human compartment in which these complex oxidative reactions are observed. The increment in oxidant chemical species leads to antioxidant depletion and consequently to the enhanced expression of pro-inflammatory genes, peroxidation of membrane lipids, depletion of nicotinamide nucleotides, enhanced intracellular Ca^2+^, cytoskeleton breakage, and DNA damage [9,36]. The most relevant research works in the literature are summarized in Table 1.

### 2.1. Cigarette Smoke Effect on Airway Diseases

Oxidative stress is involved in the pathogenesis of several respiratory diseases, including chronic obstructive pulmonary disease (COPD) and its comorbidities [38,46,47], asthma [14,48,49,50,51], cystic fibrosis [52,53], bronchiectasis [54], idiopathic pulmonary fibrosis (IPF) [55,56], and pulmonary hypertension [57]. 

Increased oxidative stress induced by tobacco smoking is a well-recognized key driving mechanism involved in the onset and development of COPD [58]. This is reflected by the elevated levels of oxidative stress markers in COPD patients [47,56,57]. Polyunsaturated fatty acids of cell membranes are the main sites of reactive compound attack, leading to lipid peroxidation processes and producing hydroperoxides, eicosanoids, and long-lived aldehydes [59]. Increased levels of aldehydic products derived from the oxidation of the cell membrane phospholipids, including malondialdehyde (MDA), acrolein, hexanal, and nonanal, were observed in the induced sputum and exhaled breath condensate (EBC) of patients with COPD compared to healthy subjects [60]. Singh and colleagues reported higher levels of MDA along with reduced activity of antioxidant enzymes, including SOD, catalase, GSH, GR, and GPx, in the blood of COPD patients. This altered oxidant/antioxidant balance was more evident in patients who smoke than non-smokers [61]. Moreover, 8-isoprostane (i.e., 8-epi-prostaglandin F2α), a marker of non-enzymatic lipid peroxidation, was increased in the sputum of COPD patients [62,63]. Cigarette smoking induced the activation of alveolar macrophages and MPO in neutrophils [64], as observed in bronchoalveolar lavage fluid (BALF) from COPD patients [46,65,66]. Moreover, the NOX pathway is involved in the airway smooth muscle remodeling observed in COPD. Indeed, an increased volume of airway smooth muscle mass was found, along with enhanced protein expression of NOX4, in the small airways of COPD patients, and the amount of NOX4 protein correlated with disease severity and inversely correlated with pulmonary function in COPD patients [67]. In addition, increased nitrogen-derived oxidative stress, nitrotyrosine, and iNOS were observed in COPD [68,69]. The equilibrium between protease and antiprotease enzymes is impaired by oxidants in the lungs, producing an imbalance in favor of proteases, such as neutrophil elastase. This imbalance facilitates the degradation of elastin present in the elastic fibers of the lung matrix, leading to the development of emphysema [59,70]. Cigarette smoke induces morphological changes in mitochondria, such as mitochondrial fission/fusion or mitophagy, contributing to age-related COPD pathogenesis [71]. Analysis of the sputum proteome and transcriptome in smokers and early-stage COPD patients showed marked xenobiotic metabolism and oxidative stress responses. Among the relevant up-regulated transcripts, there are genes that encode for glucose-6-phosphate dehydrogenase (G6PD) and phosphogluconate dehydrogenase (PGD), which contribute to the regeneration of NADPH, and therefore to the oxidative stress response [8]. Moreover, oxidant agents within cigarette smoke are able to induce, both in vitro and in vivo, changes in cystic fibrosis transmembrane conductance regulator (CFTR) gene expression, protein stability, and channel function. These dysfunctions are in common with cystic fibrosis lung disease, and play a key role in many of the pathways associated with the pathogenesis of COPD [72]. 

Positive smoking history is predictive of new-onset asthma in atopic and non-atopic subjects [47,48,49,73,74], and an increase in asthma severity, in a dose-dependent manner [50,75,76,77]. This is due to the cigarette smoke’s contribution in enhancing the oxidative stress pathways involved in asthma pathogenesis [14]. Extensive lipid peroxidation and oxidative stress mechanisms are related to asthma severity associated with active smoking. Indeed, high levels of urinary 8-isoprostane and over-expression of sputum NOX2 mRNA were observed in severe asthmatic smokers compared to severe asthmatic non-smokers [78]. Elevated levels of H_2_O_2_ were found in the exhaled breath of poorly controlled asthma patients [79]. Gene set variation analysis of severe asthma patients enrolled in the Unbiased Biomarkers for the Prediction of Respiratory Disease Outcomes (U-BIOPRED) study showed the enrichment of xenobiotic metabolism and oxidative stress pathways in current smokers compared to other groups [80]. Increased oxidative stress markers were also observed in the asthma–COPD overlap syndrome (ACOS). Indeed, ACOS patients showed a raised mitochondrial DNA/nuclear DNA ratio, which is correlated with the number of pack/years and neutrophil percentage in sputum [81].

### 2.2. Effect of NGPs on Airways

Although the deleterious effects of cigarette smoke are well established, a debate about the potential effect of NGPs on oxidative stress is still ongoing. Several in vitro and in vivo studies with different conclusions were published in recent years. Lerner and colleagues reported increased oxidative stress and inflammation biomarkers in both H292 human airway epithelial cells exposed to e-cigarette vapors for 5, 10, and 15 min, and in mice lungs exposed to e-cigarette vapors for 5 h/day for 3 days compared to air controls [82]. Increased lipid peroxidation, assessed by thiobarbituric acid reactive substance (TBARS) determination, was observed in lung homogenates from C57BL/6 mice that were exposed to e-cigarette vapor for 1.5 h, twice per day, for 2 weeks [83]. However, the in vitro and in vivo exposure settings of these studies do not reflect the standardized approach for e-cigarette aerosol generation (CRM81 regime) [84], which is central to effectively assessing the impact of e-cigarette use on human health under normal conditions of consumption. Another study evaluated the impact of e-cigarette exposure on mechanisms associated with inflammatory–oxidative responses and COPD progression in the BEAS-2B cell line and a murine model. Autophagy impairment and aggresome formation were observed in BEAS-2B cells cultured with e-cigarette aerosol-conditioned medium, and in mice exposed to e-cigarette aerosol compared to air controls [85]. However, the submerged exposure of lung cells and the aerosolization of e-cigarette liquid, without the use of a vaping machine and of a standardized regimen, are great limitations that heavily affect the results of this study. In another study, primary bronchial epithelial cells from two donors were exposed to e-cigarette vapor, with or without nicotine, and to cigarette smoke (3R4F reference cigarettes) at the air–liquid interface (ALI). Cells exposed to cigarette smoke showed levels of oxidative stress 4.5–5 times higher than those observed in e-cigarette-exposed cells [86]. However, the authors did not use standard regimes (e.g., Health Canada Intense or CRM81 regimes) [87] to generate bell- or square-shaped puff profiles. Taylor and colleagues investigated oxidative stress in H292 human bronchial epithelial cells exposed to aqueous aerosol extracts (AqE) from e-cigarette and cigarette smoke. They observed increased intracellular generation of oxidant species, a reduced GSH:GSSG ratio, and increased transcriptional activation of the nuclear factor erythroid-related factor 2 (Nrf2)-controlled antioxidant response elements (ARE), measured by a luciferase assay in H292 cells transfected with a pGL4[ARE-luc2P/Hygro] vector (Promega Corp., Madison, WI, USA), exposed to cigarette smoke AqE. Instead, no oxidative stress responses were shown after the exposure of transfected H292 cells to e-cigarette AqE [88]. Moreover, this study has some limitations. Although the smoking machine and Health Canada Intense (HCI) regime were used to generated AqEs by e-cigarette aerosols, only soluble components were captured in the AqEs, and the use of a tumor lung cell line (H292) in submerged culture may have underestimated the overall risk, albeit showing a marked effect of cigarette smoke compared to that of e-cigarette aerosols. In a recent work, mitochondrial functions were investigated in BEAS-2B exposed to total particulate matter (TPM) from a THP system and 3R4F cigarettes for 1 and 12 weeks. Similar mitochondrial dysfunctions and oxidative stress levels were found in cells when exposed to TPM concentrations from THP 20-fold higher than TPM concentrations from 3R4F cigarettes after 1-week exposure. Interestingly, the adaptation of Beas2b cells was observed after 12 weeks of exposure [89]. Moses and colleagues exposed primary bronchial epithelial cells to e-cigarette vapor (four different ECIGs from one single manufacturer) or tobacco smoke (3R4F reference cigarettes) at ALI. No cytotoxicity was observed in cells treated with e-cigarettes compared to tobacco smoke, but different gene expression levels related to xenobiotic metabolism and oxidative stress were observed both in e-cigarette- and tobacco smoke-treated cells compared to air controls. However, the magnitude of these changes was higher in cells exposed to tobacco smoke than e-cigarettes, and in the nicotine-containing e-cigarette group than the no-nicotine e-cigarette group. Moreover, similar changes in gene expression were observed in the bronchial brushing of e-cigarette users [90]. 

Many uncertainties are still to be addressed regarding chemical flavorings. A work by Jabba et al. suggests that many aldehyde flavoring compounds, including vanillin, ethyl vanillin (vanilla), and benzaldehyde (berry/fruit), react in liquids with PG and VG solvents to form chemical adducts called flavor aldehyde PG/VG acetals [91]. Assessing the effect of these chemical flavors and their corresponding acetals on bronchial (BEAS-2B) and alveolar (A549) epithelial cells, they observed that PG acetals diminished key parameters of cellular energy metabolic functions, including basal respiration, adenosine triphosphate production, and spare respiratory capacity. Despite the relevance of these findings, the results on the effects of chemical flavors from this study should be considered as partial and inconclusive, as the exposure method used did not reproduce lung cell aerosol exposure. Studies set up to evaluate the safety of these chemicals when vaped via e-cigarettes are still needed to clarify this important aspect.

The use of a variety of in vitro and in vivo models and the non-standardized puffing regimes are important issues for the evaluation of NGPs’ effects, and it is crucial to determine whether exposure conditions can be transferred to real-world situations.

**Table 1:** Relevant research on oxidative stress related to tobacco smoke and NGP aerosols in respiratory diseases.

Author	Study Findings	Product(s) Tested	Experimental Setup
Kirkham et al. [46]	Repeated low-micromolar exposure to car-bonyls (e.g., acrolein) from cigarette smoke leads to carbonyl adduct (modified pro-teins) accumulation over time in collagen type IV. Acrolein-modified proteins can ac-tivate macrophages, such as oxidative burst and the release of MCP-1, independently of other stimuli.	Tobacco smoke	In vitro
Plaschke et al. [47]	Smoking was found to be a risk factor for onset of asthma in adults.	Tobacco smoke	Human study
Rasmussen et al. [48]	Smoking is an independent risk factor for the development of asthma-like symptoms during adolescence.	Tobacco smoke	Human study
Kim et al. [49]	Active smoking may play an important role in the development of asthma and bronchial hyper-responsiveness among the elderly.	Tobacco smoke	Human study
Polosa et al. [50]	Cigarette smoking is an important predictor of asthma severity and poor asthma control.	Tobacco smoke	Human study
Polosa et al. [72]	Cigarette smoking is an important independent risk factor for the development of new asthma cases in adults with allergic rhinitis.	Tobacco smoke	Human study
Emma et al. [78]	Cigarette smoking in severe asthma patients causes greater systemic oxidative stress. Moreover, active smoking in asthmatic subjects can lead to inhibition of NOS2 mRNA expression in pulmonary cells (bronchial brushing) by negative feedback, possibly due to the high level of NO contained in cigarette smoke.	Tobacco smoke	Human study
Takahashi et al. [80]	Increased colony stimulating factor (CSF) 2 protein levels, xenobiotic metabolism, oxidative stress, and endoplasmic reticulum stress in the respiratory tract (bronchial brushing, biopsies, and sputum cells) have been observed in asthmatics who currently smoke. In former asthmatic smokers, there is a predominant neutrophilic inflammation and loss of epithelial barrier function.	Tobacco smoke	Human study
Lerner et al. [82]	Exposure to e-cigarette aerosols/juices sustains oxidative and inflammatory responses in lung cells (human airway epithelial cells and human lung fibroblasts) and pulmonary tissues in C57BL/6J mice.	NGP aerosols	In vitro
Sussan et al. [83]	Mice exposed to e-cigarette aerosol showed significantly impaired pulmonary bacterial clearance, compared to air-exposed mice. This defective bacterial clearance was partially due to reduced phagocytosis by alveolar macrophages.	NGP aerosols	In vivo
Shivalingappa et al. [85]	E-cigarette vapor exposure induces proteostasis/autophagy impairment, leading to oxidative stress, apoptosis, and senescence.	NGP aerosols	In vitro
Scheffler et al. [86]	In an in vitro model of human bronchial epithelial cells exposed to e-cigarette aerosols with different concentrations of nicotine and tobacco cigarette smoke, authors observed toxicological effects induced by smoke and aerosol, whereas the nicotine concentration did not have an effect on the cell viability.	NGPs aerosol Tobacco smoke	In vitro
Taylor et al. [88]	Concentration-dependent oxidative stress, intracellular generation of oxidant species, reduced GSH:GSSG, increased transcriptional activation of ARE, increased Caspase 3/7 activity, and strong decrease in viability were observed in human bronchial epithelial cells following exposure to cigarette smoke AqE. No cellular stress responses were detected following exposure to e-cigarette AqE.	NGP aerosols	In vitro
Malinska et al. [89]	Tobacco cigarette total particulate matter (TPM) had a stronger effect on oxidative phosphorylation, gene expression, and proteins involved in oxidative stress than TPM from a tobacco heating product (THS2.2) in a model of human bronchial epithelial cells.	NGP aerosols	In vitro
Moses et al. [90]	E-cigarette aerosol can induce gene expression changes in bronchial airway epithelium in vitro, some of which are shared with tobacco cigarette smoke. These changes were generally less pronounced than the effects of tobacco cigarette exposure and were more pronounced in e-cigarette products containing nicotine than those without nicotine.	NGP aerosols	In vitro
Jabba et al. [91]	Reaction products formed in e-liquids between flavor aldehydes and solvent chemicals have differential toxicological properties from their parent flavor aldehydes and may contribute to the health effects of e-cigarette aerosols in the respiratory systems of e-cigarette users.	NGP aerosols	In vitro

## 3. Oxidative Stress and Smoking/Vaping Related to Cardiovascular Diseases

**Figure 4:**
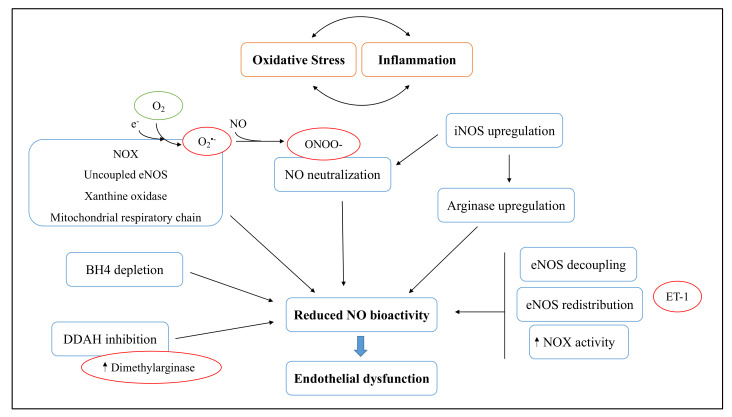
Mechanisms inducing endothelial dysfunction through reduced NO bioactivity.

Oxidative stress and inflammation derived from cigarette smoke and pollutant exposure have a prevalent role in vascular damage and endothelial dysfunction. A clear relationship was proven between smoking/pollution exposure and cardiovascular diseases, including atherosclerosis and hypertension, coronary artery disease, heart failure, peripheral arterial disease, cardiac arrhythmia or arrest, and venous thromboembolism [92]. Cardiovascular disease mortality due to the effects of smoking and pollution exceeds that for lung diseases [93]. Oxidative stress is one of the mechanisms that links the inhalation of toxicants and their cardiovascular effects, but the biological pathways of this phenomenon remain the subject of ongoing research. The endothelial dysfunction caused by oxidative stress and inflammation leads to enhanced endothelial expression of adhesion molecules, an imbalance of arachidonic acid metabolites, and increased chemoattractant molecules [94,95]. Different pro-oxidant molecules are involved in endothelial dysfunctions, including NOX enzymes, xanthine oxidase, mitochondrial enzymes, lipoxygenase, myeloperoxidase, endothelial NOS (eNOS), iNOS, and endothelin-1 (ET-1) peptide [94,95]. A crucial factor involved in endothelial function is endothelium-derived NO produced by eNOS. Under normal conditions, endothelial NO is a physiological vasodilator that exerts different vascular functions, including vasoprotection and the prevention of atherosclerosis [96]. Vascular NO inhibits platelet aggregation and adhesion to the vascular wall [97,98], and averts the platelet-derived growth factors that stimulate smooth muscle proliferation and the consequent production of matrix molecules [96]. NO also prevents the oxidative modification of LDL cholesterol and decreases the expression of chemoattractant protein MCP-1 [99] and other surface adhesion molecules that promote the development of atherosclerosis [96]. Moreover, NO has been shown to protect against a later phase of atherogenesis, inhibiting DNA synthesis, mitogenesis, proliferation, and the migration of vascular smooth muscle cells [100,101,102]. Reduced NO bioactivity by oxidative stress mechanisms is a crucial aspect of endothelial dysfunction (Figure 4). Functional eNOS transfers electrons from NADPH in the reductase domain to the heme in the oxygenase domain so that L-arginine is oxidized to L-citrulline and NO [96]. Perturbation of this electron flow led to the decoupling of oxygen reduction and NO generation with consequent superoxide production from the oxygenase domain [103]. In the presence of high levels of O_2_^•−^, NO is inactivated through the following reaction: NO + O_2_^•−^ → ONOO- [104]. Furthermore, oxidative stress contributes to NOS cofactor tetrahydrobiopterin (BH4) depletion and dimethylarginine dimethylaminohydrolase (an enzyme that degrades dimethylarginase, a potent competitive inhibitor of NOS) inhibition, with a consequent decrease in NO production [105,106]. Another factor involved in the reduction of NO bioavailability is endothelin-1 (ET-1), a potent vasoconstrictor peptide with pro-oxidant and pro-inflammatory proprieties. Indeed, ET-1 induces eNOS uncoupling and the redistribution of eNOS from the plasma membrane to the mitochondria via the phosphorylation of eNOS at Thr495 by protein kinase C δ [107]. Moreover, ET-1 overexpression enhanced NOX activity, glomerular permeability to albumin and renal inflammation, aortic vascular cell adhesion molecule 1 (VCAM-1), and monocyte/macrophage infiltration [94,108]. The inflammatory state also leads to the upregulation of iNOS. Excessive amounts of NO produced by iNOS not only contribute to ONOO- formation, but also to arginase upregulation [94]. In addition, angiotensin II signaling stimulates the upregulation and activation of NOX in the vascular wall via AT-1 receptors, contributing to oxidative stress [109,110]. As a result, positive feedback loops between oxidative stress and endothelial dysfunction/inflammation are established. The most relevant research works discussed in this section are summarized in Table 2.

### 3.1. Cigarette Smoke Effect on Endothelial Dysfunction

A large body of literature strongly indicates the deleterious cardiovascular consequences of cigarette smoking. Exposure to cigarette smoke leads to impairments of vascular tone and hemostasis, and therefore to endothelial dysfunction. Indeed, several clinical and in vitro studies suggest that cigarette smoke and its released free radicals are involved in the induction of endothelial dysfunction, mainly through NO bioavailability reduction [111,112,113]. Both active and passive cigarette smoking are associated with impairments of endothelium-dependent dilatation in a dose-dependent manner [114,115]. Moreover, it was shown that cigarette smoke is associated with uncoupling, enhanced protein expression, and reduced activity of eNOS, resulting in pro-inflammatory and oxidative stress status [112,116]. Additionally, it was shown that cigarette smoke extract (CSE) induces endothelial apoptosis by Caspase 3 activation [117]. Cigarette smoke is also able to activate the NOX pathway [118], xanthine oxidase [119], neutrophils and macrophages, and the mitochondrial respiratory chain [120], thereby contributing to oxidative stress [113]. 

ROS derived from cigarette smoke contribute to endothelial dysfunction via the activation of NF-kB, and consequently the expression of pro-inflammatory cytokines, chemokines, and adhesion molecules [121,122]. Interestingly, van den Berg and colleagues showed that NF-kB activity was higher in the peripheral blood mononuclear cells of male smokers compared to non-smokers [123]. Some evidence suggests a role for H_2_O_2_ derived from the NOX pathway in the activation of endothelial NF-kB [124,125]. 

Cigarette smoke exposure negatively influenced all stages of plaque formation, platelet activation, coagulation cascade, and anticoagulative fibrinolysis [116]. Higher content of extracellular lipids was observed in the plaques of smokers compared to those of non-smokers [126]. CSE is able to inhibit the PAF-degrading enzyme PAF acetyl-hydrolase in a dose-dependent manner [127]. Indeed, it was shown that smokers have increased circulating levels of platelet-activating factor (PAF) and PAF-like lipids [128]. Increased activity of matrix metalloproteinases (MMPs) is induced by smoking-derived oxidative stress [129], which has been implicated in the generation of an unstable plaque phenotype [130,131]. Cigarette smoke exposure resulted to be associated with increased expression of MMP-12 in plaques derived from carotid endarterectomy specimens of smokers compared to non-smokers [132]. Huang and colleagues investigated the association between cigarette smoking and cardiovascular disease-related protein biomarkers in two Swedish community-based cohorts (the Prospective Study of the Vasculature in Uppsala Seniors—PIVUS—and the Uppsala Longitudinal Study of Adult Men—ULSAM). They found 30 proteins significantly associated with current smoking in the PIVUS cohort, and 10 were replicated in the ULSAM cohort, including MMP-12, growth/differentiation factor 15 (GDF-15), urokinase plasminogen activator surface receptor (uPAR), TNF-related apoptosis-inducing ligand receptor 2 (TRAIL-R2), lectin-like oxidized LDL receptor 1 (LOX-1), hepatocyte growth factor (HGF), matrix metalloproteinase-10 (MMP-10) and matrix metalloproteinase-1 (MMP-1), endothelial cell-specific molecule 1 (ESM-1), and interleukin-27 subunit alpha (IL27-A). These protein biomarkers are associated with endothelial dysfunction, inflammation, neointimal formation, foam cell formation, and plaque instability [133].

### 3.2. Effects of NGPs on Oxidative Stress-Related Endothelial Dysfunction

Actually, there are limited and conflicting data in the literature about the potential effects of NGPs on oxidative stress related to cardiovascular risks. Teasdale and colleagues investigated gene expression by exposing human coronary artery endothelial cells (HCAEC) to aqueous filtered extracts of e-cigarette aerosol or cigarette smoke. They observed the activation of oxidant stress sensing transcription factor NFR2 and the oxidative stress pathway, and the upregulation of cytochrome p450, in HCAEC treated with CSE, but not in HCAEC treated with e-cigarette extracts. However, the use of no reference cigarette and no standard puffing regime limits their results [134]. In another in vitro study, the effects of e-cigarette AqE and CSE on human umbilical vein endothelial cells (HUVECs) were evaluated. Both CSE and e-cigarette AqE generated ROS in HUVECs after 4 h of exposure compared to controls. Moreover, DNA damage and cell death were induced by e-cigarette AqE and CSE. In all cases, the effects observed in cells treated with e-cigarette AqE were much lower than those observed after CSE exposure [135]. In a cross-over single-blind study, Carnevale and colleagues evaluated oxidative stress biomarkers in non-smokers and chronic tobacco smokers after either tobacco smoking or e-cigarette vaping. Both acute exposure to tobacco smoking and e-cigarette vapor increased oxidative biomarkers, as evidenced by increased levels of sNox2-dp (marker of NOX activation) and 8-isoPGF2α, and decreased NO bioavailability, vitamin E levels, and FMD. Nevertheless, the effects of e-cigarettes were less pronounced than those caused by cigarettes, particularly with regard to the levels of sNox2-dp, 8-isoPGF2α, and NO bioavailability [136]. An increase in sympathetic tone and LDL oxidizability was reported in e-cigarette users compared to non-user controls, but no changes were observed for indicators of systemic oxidative stress (C-reactive protein and fibrinogen) between the two analyzed groups. The differential effects of an e-cigarette vehicle (propylene glycol and glycerol) and nicotine on microcirculatory function, arterial stiffness, hemodynamic parameters, and oxidative stress were evaluated in a randomized cross-over single-blind trial. Twenty-five tobacco smokers were exposed to vaping with and without nicotine, and sham vaping (with the e-cigarette turned off) under specific vaping conditions (25 puffs with puff duration of 4 s every 30 s). No modifications of cardiovascular parameters or oxidative stress were observed with sham vaping and no-nicotine vaping. Instead, vaping with nicotine resulted in the modification of cardiovascular parameters and increased plasma myeloperoxidase [137]. Thus, based on these results, it seems that e-cigarettes’ effects on cardiovascular and oxidative stress are due to nicotine and not to high-temperature e-cigarette vehicle vaporization. In another clinical study, Ikonomidis and colleagues evaluated the acute and chronic effects of vaping on aortic stiffness (pulse wave velocity and augmentation index) and oxidative stress (malondialdehyde plasma concentrations). They showed that vaping and smoking affected arterial elasticity and oxidative stress parameters when measured after 7 min of smoking/vaping at baseline. Meanwhile, reduced central and brachial systolic blood pressure, arterial wave reflections, and oxidative stress were observed in smokers who switched to e-cigarettes within 1 month [138].

Recently, Espinoza-Derout and colleagues showed the detrimental cardiovascular and cardiac effects of e-cigarettes with nicotine in an e-cigarette exposure model of apolipoprotein-E knockout (ApoE^−^/^−^) mice. Mice were intermittently exposed to saline aerosol, nicotine-free e-cigarette aerosol (e-cigarette (0%)), and e-cigarette with 2.4% nicotine (e-cigarette (2.4%)) aerosol for 12 weeks. Treatment with the nicotine e-cigarette (2.4%) showed reduced left ventricular fractional shortening and ejection fraction, a change in genes associated with metabolism, circadian rhythms, and inflammation, ultrastructural abnormalities indicative of cardiomyopathy in cardiomyocytes, increased oxidative stress, and increased atherosclerotic lesions, compared to mice treated with the e-cigarette without nicotine and saline [139].

Kuntic and colleagues studied the effects of short-term e-cigarette vapor exposure (1, 3, or 5 days) on 124 C57BL/6 mice (male, aged 12 ± 3 weeks) and 27 null Nox2 mice (background C57BL/6; male, aged 13 ± 3 weeks) for 2 h/day (6 times 20 min). For the exposure, they used unflavored e-cigarette liquids with and without nicotine. The scientists concluded that exposure to e-cigarette vapors induces inflammation and oxidative stress in vascular and cerebral tissue, and increases blood pressure in mice, hypothesizing a NOX-2-driven mechanism for vascular oxidative stress and inflammation, with an important role of ET-1 and FOXO-3 signaling. Finally, they established that the aldehydes produced during vaporization were responsible for the production of NOX-2-driven oxidative stress and inflammation [140,141]. However, the exposure method of mice used in this study was not standardized, which leads to questionable results. Moreover, the final observation by the scientists underlines the fact that the quality of e-cigarette devices and liquids is fundamental in determining the pro-inflammatory and oxidative stress effects of vapors. In fact, as observed by Farsalinos et al., aldehyde emission in the vapor of e-cigarettes is closely related to the characteristics and settings of the devices, liquids, and to the laboratory setting [140]. In light of these observations, the results of Kuntic and colleagues can be widely questioned as a function of the characteristics of the experimental settings. Moreover, Lee and colleagues demonstrated that the cytotoxicity of e-liquids varied considerably, with the cinnamon-flavored product being more potent and leading to significantly reduced cell viability, increased levels of reactive oxygen species (ROS), activity of Caspase 3/7, and absorption and activation of low-density lipoproteins of the pathway related to oxidative stress [142].

**Table 2:** Relevant research on oxidative stress related to tobacco smoke and NGP aerosols in cardiovascular diseases.

Author	Study Findings	Product(s) Tested	Experimental Setup
Barua et al. [112]	The study confirmed that oxidative stress plays a central role in smoking-mediated dysfunction of NO biosynthesis in human coronary artery endothelial cells.	Tobacco smoke	In vitro
Celermajer et al. [114]	Both active and passive smoking impaired endothelium-dependent arterial dilatation, suggesting early arterial damage in healthy young adults.	Tobacco smoke	Human study
Zeiher et al. [115]	The impairment of coronary arterial vasodilator function is associated with long-term cigarette smoking.	Tobacco smoke	Human study
Raveendran et al. [117]	Cigarette smoke extract induced human aortic endothelial cells’ (HAECs) apoptosis, but endogenous NO production reduced the cigarette smoking-induced apoptosis.	Tobacco smoke	In vitro
Jaimes et al. [118]	Thiol-reactive stable compounds in cigarette smoke increase endothelial O_2_^•−^ through NADPH oxidase activation, thereby reducing NO bioactivity and resulting in endothelial dysfunction.	Tobacco smoke	In vitro
Kayyali et al. [119]	Cigarette smoke condensate induced xanthine oxidase mRNA expression and xanthine oxidase gene promoter activity.	Tobacco smoke	In vitro
Talukder et al. [120]	Exposure of C57BL/6J mice to cigarette smoke for 32 weeks led to blunted weight gain, hypertension, endothelial dysfunction, leukocyte activation with ROS generation, decreased NO bioavailability, and mild cardiac hypertrophy.	Tobacco smoke	In vivo, mouse model
van den Berg et al. [123]	The transcription factor NF-kB was increased in the peripheral blood mononuclear cells of smokers compared to non-smokers, confirming the role of this biomarker in smoke-induced inflammation.	Tobacco smoke	Human study
Orosz et al. [124]	Results from this study suggest that water-soluble components of cigarette smoke activate the vascular NAD(P)H oxidase with increased production of O_2_^•−^ and consequently H_2_O_2_. NAD(P)H oxidase-derived H_2_O_2_ activates NF-kB, leading to pro-inflammatory alterations in vascular phenotype.	Tobacco smoke	In vivo, rat model
Miyaura et al. [127]	Cigarette smoke extract inhibits, in a dose-dependent manner, the activity of PAF-acetylhydrolase, an important enzyme that regulates the degradation of the vascular pro-inflammatory platelet-activating factor (PAF).	Tobacco smoke	In vitro–ex vivo
Imaizumi et al. [128]	The platelet-activating factor-like lipid(s) (PAF-LL) were detected in LDL and HDL plasma lipoproteins, and their levels were significantly increased in smokers after smoking, contributing to atherosclerosis.	Tobacco smoke	Human study
Kangavari et al. [132]	Cigarette smoking increases markers of inflammation, including macrophage immunoreactivity (CD68 expression) and MMP-12, and tissue destruction in atherosclerotic plaques (TIMP-1) in smokers compared to non-smokers.	Tobacco smoke	In vitro–ex vivo
Huang et al. [133]	Active cigarette smoking status was positively associated with increased matrix metalloproteinase-12 (MMP-12), growth/differentiation factor 15 (GDF-15), urokinase plasminogen activator surface receptor (uPAR), TNF-related apoptosis-inducing ligand receptor 2 (TRAIL-R2), lectin-like oxidized LDL receptor 1 (LOX-1), hepatocyte growth factor (HGF), matrix metalloproteinase-10 (MMP-10), and matrix metalloproteinase-1 (MMP-1). Negative association with active smoking was reported for endothelial cell-specific molecule 1 (ESM-1) and interleukin-27 subunit alpha (IL27-A). All these results suggest the interference of smoking with the atherosclerosis process.	Tobacco smoke	Human study
Teasdale et al. [134]	Cigarette smoke extract induced the activation of NRF2 and upregulation of cytochrome p450 in human coronary artery endothelial cells. However, e-cigarette extract did not induce NRF2 nuclear activation, or the upregulation of cytochrome p450.	NGP aerosols	In vitro
Anderson et al. [135]	E-cigarette aerosol induced reactive oxygen species, DNA damage, and cell death in human umbilical vein endothelial cells. However, the effects of e-cigarette aerosols were lower than those of cigarette smoke applied at the same nicotine concentration.	NGP aerosols	In vitro
Carnevale et al. [136]	Significant increase in soluble NOX2-derived peptide and 8-iso-prostaglandin F2α and a significant decrease in nitric oxide bioavailability, vitamin E levels, and FMD were observed in e-cigarette (dual users) and traditional cigarette consumers. However, e-cigarettes seemed to have a lesser impact.	NGP aerosols	Human study
Chaumont et al. [137]	Sham vaping and vaping without nicotine were not associated with modification of cardiovascular parameters or oxidative stress. Instead, vaping with nicotine resulted in modification of cardiovascular parameters and increased plasma myeloperoxidase.	NGP aerosols	Human study
Ikonomidis et al. [138]	Aortic stiffness, assessed by pulse wave velocity (PWV) and augmentation index (AIX75), exhaled CO concentration, and oxidative stress, assessed by malondialdehyde (MDA) plasma concentrations, were reduced in smokers who switched to e-cigarettes after 1 month of use.	NGP aerosols	Human study
Espinoza-Derout et al. [139]	E-cigarette with 2.4% nicotine decreased left ventricular fractional shortening and ejection fraction, induced changes in genes associated with metabolism, circadian rhythm, and inflammation, and also induced ultrastructural abnormalities of cardiomyocytes in ApoE−/− mice compared to controls (saline). Additionally, increased oxidative stress and mitochondrial DNA mutations were observed in mice treated with e-cigarettes (2.4%).	NGP aerosols	In vivo, mouse model
Farsalinos et al. [140]	The release of toxic aldehydes is associated with the generation of dry puffs. Under realistic conditions, e-cigarettes emit minimal aldehydes/g liquid at both low and high power.	NGP aerosols	In vitro
Kuntic et al. [141]	E-cigarette vapor exposure, particularly acrolein, increases vascular, cerebral, and pulmonary oxidative stress via a NOX-2-dependent mechanism in mice.	NGP aerosols	In vivo, mouse model
Lee et al. [142]	Exposure to cinnamon-flavored e-cigarette vapor led to significantly decreased cell viability, increased reactive oxygen species (ROS) levels, Caspase 3/7 activity, low-density lipoprotein uptake, activation of oxidative stress-related pathway, and impaired tube formation and migration, confirming endothelial dysfunction.	NGP aerosols	In vitro

## 4. Oxidative Stress and Smoking/Vaping Related to Tumors

**Figure 5:**
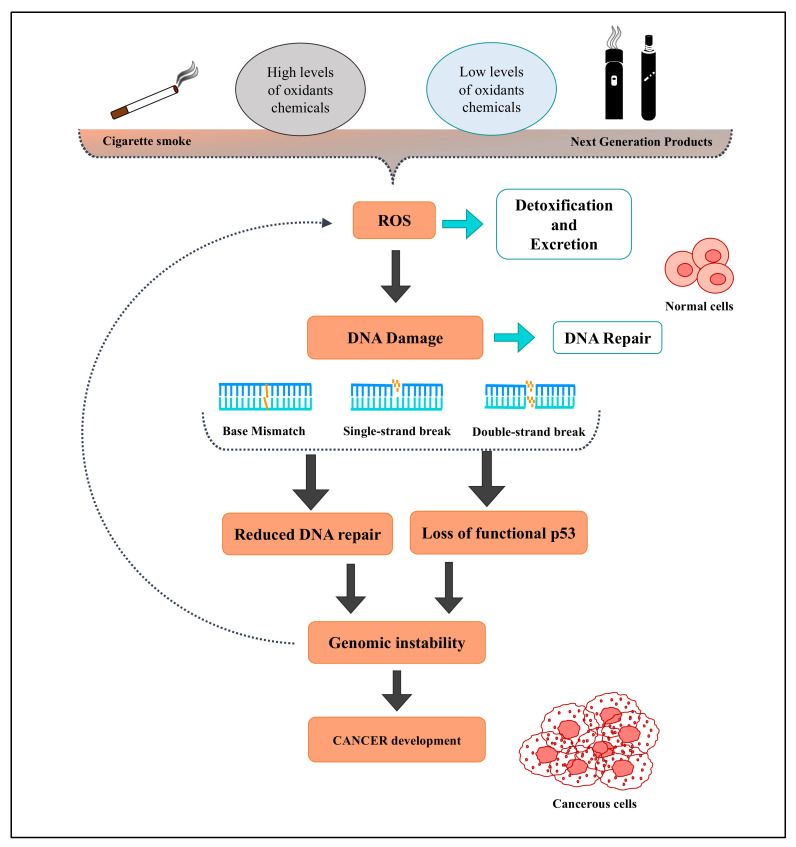
The role of reactive oxygen species (ROS) in the development of cancer. The increase in ROS in normal cells triggers stress responses and DNA repair to repair the ROS-mediated damage to genetic materials. However, the exposure to high levels of oxidants and the consequential redox imbalance lead to DNA damage, including base mismatch, single-strand break, or double-strand break. Moreover, ROS induce DNA mutations that could cause a loss of p53 function and DNA repair disfunction, leading to genomic instability, which further leads to the activation of oncogenes, aberrant metabolic stress, mitochondrial dysfunction, and a decrease in antioxidants. All these events activate a vicious cycle that amplifies oxidative stress and promotes cancer development.

It is well established that excessive ROS/RNS production damages cellular components, such as DNA, proteins, and lipids, and alters nuclear or mitochondrial DNA, thereby causing carcinogenesis processes [143]. An overview of these mechanisms is shown in Figure 5. The first evidence that associated oxidative stress and cellular transformation was presented in 1981 by Oberley, who observed that insulin increased intracellular H_2_O_2_ levels and enhanced tumor cell proliferation [144]. Moreover, it was observed that cancer cells are more oxidatively stressed compared to their non-tumorous counterparts [145]. An important role in cancer cell proliferation is performed by mitochondria, which produce an abundant amount of ATP for the synthesis of lipids, proteins, and nucleotides. Oxidative perturbation can cause mitochondrial dysfunction and cell cycle arrest [146]. Nevertheless, the role of ROS in cancer mechanisms remains controversial. Indeed, the possible oncogenic or tumor-suppressive roles of ROS were evaluated under various conditions in several studies [146]. In the initial phase of carcinogenesis, ROS may induce gene mutation and structural alteration to DNA. Meanwhile, in the evolving phase, ROS are involved in the promotion of abnormal gene expression, blockage of cellular communication, and modification of second-messenger systems. Cell proliferation and differentiation are stimulated by a moderate increase in ROS levels. Instead, excessive ROS formation overcomes the cellular antioxidant capacity and triggers cell death [147,148]. In cancer cells, the aberrant proliferation leads to increased ROS generation, which is counteracted by the upregulation of antioxidant mechanisms, especially at the early stages of tumor development [149,150,151]. Low–moderate levels of ROS are able to stimulate numerous factors associated with tumor cell growth and survival, including the phosphorylation of mitogen-activated protein kinase (MAPK) and extracellular signal-regulated kinase (ERK), cyclin D1 expression, and JUN N-terminal kinase (JNK) [152,153,154,155,156]. Furthermore, ROS can interact with the redox-sensitive cysteine residues of some tumor suppressor factors (phosphatase and tensin homolog—PTEN—and protein tyrosine phosphatases—PTPs), making them inactive [152,153,154,155].

The results of these mechanisms are the increase in cell proliferation and/or decrease in apoptosis, with the consequent development of tumors. The most relevant research works discussed here are summarized in Table 3.

### 4.1. Cigarette Smoke Effects on Cancer Development

Overall, 30% of all cancer deaths are caused by cigarette smoking [156]. The first evidence of the link between cigarette smoking and cancer was observed late in the 1930s, and the causal association with lung cancer was established in the 1950s [157,158]. In the following years, the evidence of the cigarette smoking/cancer development relationship increased considerably. Indeed, in 1985, the international working group of the International Agency for Research on Cancer (IARC) established the causal relation between tobacco smoking and several malignancies in different sites of the human body, including the lungs, oral cavity, pharynx, larynx, pancreas, urinary bladder, renal pelvis, and urethra [159]. Subsequently, the list of smoking-related cancers became longer, with the addition of cancer of the nasal cavities and nasal sinuses, esophagus, stomach, liver, kidney, uterine cervix, and bone marrow (myeloid leukemia) [160]. In a metanalysis based on the 2004 IARC Monograph on Tobacco Smoke and Involuntary Smoking, the risk magnitude was evaluated for 13 cancer sites. The highest relative risk (RR) for current smokers was found for lung cancer, followed by laryngeal, pharyngeal, digestive tract, and oral cancers [161]. 

The central role of tobacco smoke/free radicals in the development of cancer has been observed in several studies [162,163,164]. As stated previously, both the tar and gas phase of cigarette smoke contain stable oxidants with a very long half-life, including semiquinones (QH•) and carbon-centered radicals [165]. Moreover, NO is implicated in carcinogenesis and tumor promotion. Indeed, the cigarette smoke induction of iNOS leads to NO-mediated DNA damage and DNA repair mechanisms. Moreover, the expression of iNOS is positively associated with p53 mutation in tumors of the colon, lung, and oropharynx [166]. Interaction between cigarette smoke oxidants and DNA leads to the formation of oxidized DNA bases such as 8-oxo-7,8-dihydroguanine (8-oxoGua) and 8-oxo-2′-deoxyguanosine (8-oxodG) [165,166,167]. The latter is a biomarker to quantify oxidative DNA damage in the initial phases of carcinogenesis [168,169]. Higher levels of 8-hydroxydeoxyguanosine (8-OHdG) expression and secretion were observed in smokers than non-smokers with and without lung cancer. Moreover, the levels of 8-OHdG in BALF were associated with the TNM stage according to the TNM classification of malignant tumors [170]. Changes in miRNA expression and proteome were observed after long-term cigarette smoke exposure in an in vitro study by Advani and colleagues. After 12 months of cigarette exposure, 112 upregulated and 147 downregulated miRNAs were detected in non-small lung cancer cells (H292 cell line). Moreover, 3959 proteins were differentially expressed in cigarette smoke-treated H292 cells related to phagosome maturation, the antigen presentation pathway, the nuclear factor erythroid 2-related factor 2-mediated oxidative stress response, and cholesterol biosynthesis pathways [171]. 

### 4.2. Effects of NGPs on Oxidative Stress-Related Carcinogenesis

Several studies indicated that NGP vapors contain much lower carcinogenic and toxic substances compared to cigarette smoke [172,173,174,175,176]. However, a small percentage of chemical compounds, such as impurities, volatile organic compounds, and flavor degradation products, may be present in the NGP vapors.

Nevertheless, conflicting data about NGPs’ effects on oxidative stress-related carcinogenesis are present in the literature. Ganapathy and colleagues exposed human epithelial normal bronchial cells to e-cigarette vapor and tobacco smoke extracts. A nicotine-independent but dose-dependent increase in DNA damage (8-oxo-dG) was observed in cells treated with e-cigarette AqE, but these levels were lower than those observed in cells treated with CSE. Moreover, they examined the mechanisms that modulate DNA damage in cells treated with e-cigarette AqE, identifying an increase in cellular ROS, a decrease in total antioxidant capacity, and a decrease in the expression of proteins essential for DNA damage repair [177]. In a similar study, the effects of e-cigarette extract were investigated in a panel of cell lines (HaCaT, UMSCC10B, and HN30). Increased DNA damage, arrest in G1 and G2, increased apoptosis, necrosis, and cell death were observed after exposure to e-cigarette extracts. However, in both studies, the submerged exposures of airways cells do not represent the ideal setting for this kind of study. The exposure of airway cells at ALI would have been a better system. In another in vitro study, Breheny and colleagues assessed the toxicological and biological responses of two THPs and a reference cigarette (3R4F). Mutagenicity, genotoxicity, cytotoxicity, tumor promotion, and oxidative stress in vitro showed significantly reduced responses in cells treated with THPs compared to 3R4F [178].

Tang and colleagues showed that FVB/N mice exposed for 54 weeks to e-cigarette smoke suffered from extensive DNA damage in the lungs, heart, and bladder mucosa and reduced DNA repair in the lungs. Indeed, products derived from e-cigarette vapor, such as nicotine and its derivatives, cause the same deleterious effects in human lung epithelial cells and bladder urothelial cells [179]. 

Canistro and colleagues showed that e-cigarettes have a potent booster effect on phase I carcinogen bioactivation enzymes, including polycyclic aromatic hydrocarbon (PAH) activators, and increase oxygen free radical production and DNA oxidation to 8-hydroxy-2′-deoxyguanosine [180].

Another research group led by Cirillo and colleagues showed that, by fixing the voltage of conventional 3.5 V electronic cigarettes, the amount of the selected aldehydes increased as the resistance changed to 1.5 and 0.25 Ω. Under these conditions, they exposed Sprague Dawley rats to e-cigarette aerosol for 28 days and assessed the lung inflammation status, oxidative stress, tissue damage, and homeostasis of the blood. The results obtained showed that there was a variation in antioxidant and phase II enzymes, probably related to the increase in ROS levels due to the enhanced xanthine oxidase and monooxygenases linked to P450. Furthermore, the scanning electron microscope frames showed disorganization of the alveolar and bronchial epithelium in the 0.25 Ω group [181].

In a recent interesting study by Song et al., the authors assessed inflammatory cells and cytokines, genome-wide gene expression, and DNA methylation in bronchial biopsies, bronchial brushing, and BAL from never smokers, smokers, and e-cigarette users. Despite the limitations in the selection of vaping subjects, including a combination of both ex-smokers and naïve vapers, as well as not discussing the nicotine medium content that they vaped or the settings of the e-cigarettes that they used, the findings of the researchers in this cross-sectional study suggest that smoking’s effects on the lungs may be at least partially reversible in smokers who switch to e-cigarettes. These findings need to be substantiated in longitudinal studies, including randomized trials [182]. 

The safety profile of NGPs in cancer needs further studies, especially clinical studies, to better understand the toxicological effects of these products in promoting carcinogenesis.

**Table 3:** Relevant research on oxidative stress related to tobacco smoke and NGP aerosols in cancer.

Author	Study Findings	Product(s) Tested	Experimental Setup
Leanderson et al. [162]	This study demonstrates the ability of cigarette smoke condensate to generate hydrogen peroxide and to hydroxylate deoxyguanosine (dG) residues in isolated DNA from calf thymus to 8-hydroxydeoxyguanosine (8-OHdG). It seems that hydroquinone and catechol may be responsible for the ability of cigarette smoke to cause 8-OHdG formation in DNA, and that the oxidative DNA damage is due to the action of hydroxyl radicals formed during the dissociation of hydrogen peroxide. Moreover, the hydrogen peroxide in cigarette smoke is generated via the autooxidation of hydroquinone and catechol.	Tobacco smoke	In vitro
Asami et al. [163]	Cigarette smoking induces an increase in oxidative DNA damage, 8-hydroxydeoxyguanosine in human lung, obtained by surgical lobectomy or pneumonectomy.	Tobacco smoke	Human study
Huang et al. [164]	This study showed that cigarette combustion will produce a high concentration of ROS and they are mainly in the gaseous phase of smoke (PM2.5). These ROS come from the combustion process and not from the tobacco leaves. There is no effective means of eliminating ROS from mainstream smoke, regardless of whether a cigarette filter contains active charcoal.	Tobacco smoke	In vitro
Valavanidis et al. [165]	Results from this work show that aqueous cigarette tar (ACT) solutions can generate adducts with DNA nucleobases, particularly the mutagenic 8-hydroxy-2′-deoxyguanosine. Moreover, synergistic effects in the generation of HO^•^ with environmental respirable particles (asbestos fibers, coal dust, etc.) and ambient particulate matter (PM), such as PM(10), PM(2.5), and diesel exhaust particles (DEP), were observed. It seems that the semiquinone radical system has the potential for redox recycling and oxidative action.	Tobacco smoke	In vitro
Cao et al. [170]	In this study, authors reported a higher level of 8-OHdG expression and secretion in airways of lung cancer patients than that of non-cancer controls; 8-OHdG expression was associated with the TNM stage. Additionally, cigarette smoke-induced oxidative DNA damage response was observed in bronchial epithelial cells in vitro and in vivo. These findings underline the importance of smoking in oxidative DNA damage response of lung cancer patients and also suggest 8-OHdG as a potential diagnostic biomarker for lung cancer.	Tobacco smoke	Human study/in vitro study
Advani et al. [171]	This study reports original observations on long-term (12 months) cigarette smoke effects in the H292 cell line, on miRNA expression profiling, and quantitative proteomic analysis. Authors identified 112 upregulated and 147 downregulated miRNAs (by twofold) in cigarette smoke-treated H292 cells. Moreover, they identified 303 proteins overexpressed and 112 proteins downregulated (by twofold). Moreover, 39 miRNA target pairs (proven targets) were differentially expressed in response to chronic cigarette smoke exposure. Gene ontology analysis of the target proteins revealed enrichment of proteins in biological processes driving metabolism, cell communication, and nucleic acid metabolism. Pathway analysis revealed the enrichment of phagosome maturation, antigen presentation pathway, nuclear factor erythroid 2-related factor 2-mediated oxidative stress response, and cholesterol biosynthesis pathways in cigarette smoke-exposed cells.	Tobacco smoke	In vitro
Goniewicz et al. [172]	E-cigarettes deliver nicotine by an aerosol, which was found to contain some toxic substances (carbonyls, volatile organic compounds, nitrosamines, and heavy metals). However, the levels of toxicants were 9–450 times lower than in cigarette smoke and were, in many cases, comparable with trace amounts found in the reference product (a medicinal nicotine inhaler; Nicorette^®^ inhalator) and in blank samples.	NGP aerosols	In vitro
Takahashi et al. [173]	This study demonstrates that emission levels for selected cigarette smoke constituents, so-called “Hoffmann analytes”, and in vitro toxicity (measured by in vitro bacterial reverse mutation, micronucleus and neutral red uptake assays) of aerosol from a novel tobacco vapor product (NTV) were substantially lower than in 3R4F cigarette smoke or absent. The authors did not detect any measurable genotoxicity or cytotoxicity.	Tobacco smoke NGP aerosols	In vitro
Schaller et al. [174]	The chemical composition, in vitro genotoxicity, and cytotoxicity of the mainstream aerosol from the tobacco heating system 2.2 (THS2.2), a THP, were compared with those of the mainstream smoke from the 3R4F reference cigarette. The aerosol from THS2.2, compared with 3R4F smoke, showed a significant reduction of more than 90% for the majority of the analyzed harmful and potentially harmful constituents (HPHCs), while the mass median aerodynamic diameter of the aerosol remained similar, even under extreme puffing regimen. A reduction of around 90% was also observed when comparing the cytotoxicity determined by the neutral red uptake assay and the mutagenic potency in the mouse lymphoma assay. The THS2.2 aerosol was not mutagenic in the Ames assay. When using puffing regimens that were more intense than the standard Health Canada Intense (HCI) machine smoking conditions, the HPHC yields remained lower than when smoking the 3R4F reference cigarette with the HCI regimen.	NGP aerosols	In vitro
Jaccard et al. [175]	In this study, it is demonstrated that the aerosol from a THP, the tobacco heating system 2.2 (THS2.2), has a mean reduction of around 90% on average across a broad range of harmful and potentially harmful constituents (HPHC) compared against the levels of HPHC of cigarettes representative of selected markets, well in line with the reduction observed against 3R4F reference cigarette smoke constituents in previous studies.	NGP aerosols Tobacco smoke	In vitro
Saffari et al. [176]	In this study, particles generated by e-cigarettes showed a 10-fold decrease in the total emission of particulate elements compared to normal cigarette smoke. Nevertheless, specific metals (e.g., Ni and Ag) displayed a higher emission rate from e-cigarette devices (not from e-liquid). Organic species in e-cigarette aerosol showed lower emission rates compared to tobacco cigarette smoke. Moreover, polycyclic aromatic hydrocarbons (PAHs) from e-cigarette aerosol were non-detectable, while substantial emission of these species was observed from tobacco cigarettes.	NGP aerosols Tobacco smoke	In vitro
Ganapathy et al. [177]	This study shows that exposure of human oral and lung epithelial cells to e-cigarette aerosol extracts suppressed the cellular antioxidant defenses and led to significant DNA damage. Overall, e-cigarette aerosol extracts induced significantly less DNA damage than mainstream smoke extracts, as measured by q-PADDA. However, the levels of oxidative DNA damage were similar or slightly higher after exposure to e-cigarette aerosol compared to mainstream smoke extracts.	NGP aerosols Tobacco smoke	In vitro
Breheny et al. [178]	This study assessed the toxicological and biological responses of aerosols from both hybrid and heated tobacco products (HTPs) using in vitro test methods, which were outlined as part of a framework to substantiate the risk reduction potential of novel tobacco and nicotine products. All the THPs tested demonstrated significantly reduced responses in in vitro assays (evaluating mutagenicity, genotoxicity, cytotoxicity, tumor promotion, oxidative stress, and endothelial dysfunction) when compared to 3R4F tobacco cigarette smoke.	NGP aerosols Tobacco smoke	In vitro
Tang et al. [179]	In this study, it was found that mice exposed to e-cigarette aerosol for 54 weeks developed lung adenocarcinomas (9 of 40 mice, 22.5%) and bladder urothelial hyperplasia (23 of 40 mice, 57.5%).	NGP aerosols	In vivo, mouse model
Canistro et al. [180]	This study demonstrates the co-mutagenic and cancer-initiating effects of e-cigarette aerosol in a rat lung model. The authors found that e-cigarettes have a powerful booster effect on phase I carcinogen-bioactivating enzymes, including activators of polycyclic aromatic hydrocarbons (PAHs), and increase oxygen free radical production and DNA oxidation to 8-hydroxy-2′-deoxyguanosine.	NGP aerosols	In vivo, rat model
Cirillo et al. [181]	This study showed that the manipulation of e-cig resistance influences the carbonyl production from non-nicotine vapor and the oxidative and inflammatory status in a rat model. Sprague Dawley rats were exposed to e-cig aerosol generated under a voltage setting of 3.5 with different resistances from 1.5 to 0.25 Ohm for 28-days; the authors found a perturbation of the antioxidant and phase II enzymes and a disorganization of alveolar and bronchial epithelium in 0.25 Ohm group.	NGP aerosols	In vivo, rat model
Song et al. [182]	In this study, authors conducted a cross-sectional analysis of bronchoalveolar lavage and bronchial brushings from 73 subjects (42 never smokers, 15 e-cig users, and 16 smokers). Lung inflammation (by cell counts), cytokines, genome-wide gene expression, and DNA methylation were assessed. Inflammatory cell counts and cytokines from the e-cigarette users showed values intermediate between smokers and never smokers, with levels for most of the biomarkers more similar to never smokers. For differential gene expression (for smoking-related pathways) and DNA methylation, e-cigarette users were also more similar to never smokers.	NGP aerosols Tobacco smoke	In vivo

## 5. Conclusions

The impact of tobacco cigarette smoke on oxidative stress signaling in respiratory diseases is clear, as with COPD and asthma. The safety profile of NGPs, including electronic cigarettes and tobacco heating products, seems to be higher than that of tobacco cigarettes, but further studies are needed to better understand the toxicological effects of these products with long-term exposure. Despite the relevance of the findings reported in this literature review, some results are of concern regarding experimental and procedural limitations, such as the exposure methods of in vitro studies. Moreover, studies on the safety of the chemical flavorings when vaped are still needed to clarify this important aspect.

The use of a variety in vitro and in vivo models and the non-standardized puffing regimes are important issues for the evaluation of NGPs’ effects, and it is crucial to determine whether and how exposure conditions can be transposed to real-world situations. 

To date, the scientific evidence available constitutes a puzzle of information, which, despite the various limitations discussed, already shows a reduced impact of ENDS on oxidative stress compared to cigarette smoking, even if they do not completely eliminate it. This means that the subject remains debated since, however, a negative effect on cellular health is also induced by ENDS. We can therefore state that these devices represent a promising harm reduction tool, but not a harm eradication tool. Above all, we do not yet possess a full understanding of the extent of the harm reduction of ENDS compared to the tobacco cigarette.
